# Organoselenium Compounds Derived from Natural Metabolites

**DOI:** 10.3390/ph18111749

**Published:** 2025-11-17

**Authors:** Agata J. Pacuła-Miszewska, Magdalena Obieziurska-Fabisiak, Jacek Ścianowski

**Affiliations:** 1 Department of Toxicology, Faculty of Pharmacy, Medical University of Gdańsk, Al. Gen. J. 107 Hallera Street, 80-416 Gdansk, Poland; 2 Department of Organic Chemistry, Faculty of Chemistry, Nicolaus Copernicus University, 7 Gagarin Street, 87-100 Torun, Polandjsch@umk.pl (J.Ś.)

**Keywords:** selenium, natural metabolites, antioxidants, reagents, catalysts

## Abstract

**Background/Objectives**: Natural metabolites, due to their abundance, structural diversity, and availability in enantiomerically pure form, are broadly utilized in the synthesis of reagents, catalysts, building blocks, and potential therapeutics. To date, various organoselenium compounds, including selenides, diselenides, selenols, selenonium salts, and ylides, have been created based on the scaffold of primary and secondary metabolites like amino acids, sugars, nucleic bases, terpenes, and steroids. Their synthesis and application routes as reagents and catalysts in organic synthesis and biological systems are summarized in the presented review. **Methods**: The gathered material has been divided into two sections—naturally derived organoselenium compounds, such as antioxidants and GPx-mimetics, and reagents utilized in modern organic transformations. **Results**: The review summarizes the utility of natural scaffolds in the construction of organoselenium compounds with promising applications as antioxidant-type catalysts in biological systems (GPx-mimetics) and potent reagents for organic transformations, including asymmetric reactions. **Conclusions**: This review provides a comprehensive overview of known organoselenium reagents derived from natural compounds, discusses the advantages of their use in medicinal chemistry and modern organic synthesis, and outlines prospective directions for future development in this area.

## 1. Introduction

Natural evolution is a one-of-a-kind phenomenon that continuously selects and modifies chemical structures to fit a specific biochemical process perfectly. The created metabolites enable the living cell to grow, reproduce, and survive in the surrounding environment. Naturally occurring molecules can be divided into those essential for survival, like amino acids, sugars, fatty acids, and nucleic bases (primary metabolites), and those involved in interacting with the surrounding environment, e.g., terpenes and alkaloids (secondary metabolites) [1,2]. The characteristic features of these compounds—structural diversity, fixed stereochemistry, and facile availability from recyclable sources—are highly desired properties in chemical syntheses dealing with the design of reagents, catalysts, or derivatives of medicinal value.

Interest of the scientific community in organoselenium chemistry has grown with the successful application of organoselenium compounds (OSeCs) in medicinal chemistry and material science and as reagents and catalysts in modern organic transformations [3,4,5]. The current development of green and environmentally friendly protocols for their preparation is an additional advantage when considering further extension of the scope of known OSeCs [6].

**Scheme 1:**
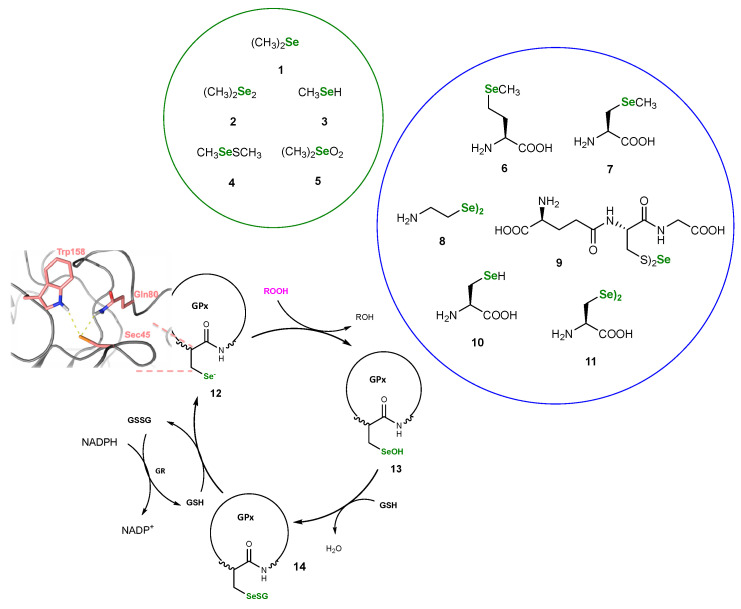
Naturally abundant organoselenium compounds **1**–**12**.

Selenization of natural scaffolds to modify and improve the reactivity of metabolites is currently a promising trend in organoselenium chemistry [7]. The selenium atom exists in nature, in the animal and plant kingdoms, and in both organic and inorganic forms [8,9,10]. As selenate (SeO_4_^2−^) and selenite (SeO_3_^2−^), which are highly soluble in soil, they are absorbed by plants and transformed into simple compounds like dimethyl selenide **1**, dimethyl diselenide **2**, methylselenol **3**, dimethyl selenyl sulfide **4,** and methyl selenone **5**. Additionally, plants can convert mineral selenium into Se amino acids like selenomethionine (SeMet) **6** and methylselenocysteine (MSC) **7**. Its presence in soil and plants is crucial for proper dietary intake as it is an essential micronutrient for animals and humans. Several Se-derivatives are necessary for important biochemical processes to occur. Selenocystamine **8** catalyses O2-mediated oxidation of an excess of glutathione to its disulfide, enabling protein folding. Selenodiglutathione **9** reduces oxidised thioredoxin in the thioredoxin/oxidoreductase dithiol-disulfide system. Finally, the 21st amino acid, selenocysteine (Sec) **10**, along with its metabolites like diselenide **11** and methylated form **7**, can be considered important biocatalysts that help maintain redox homeostasis at physiological levels. Sec **10** builds up the active side of the antioxidant Se-enzyme glutathione peroxidase (GPx). Highly reactive selenide anion RSe^−^ **12** eliminates the excess of H_2_O_2_ according to the GPx-catalytic cycle presented in Scheme 1 [11]. The active selenol group **12** is initially oxidised to form selenenic acid **13**. This intermediate then reacts with glutathione (GSH), leading to the formation of selenenyl sulfide **14**. The original selenol **12** is regenerated through a subsequent reaction between compound **14** and another glutathione molecule, during which oxidized glutathione (GSSG) is released.

Besides their important biochemical role, the naturally derived organoselenium compounds have also been found to have applications in chemical synthesis and medicinal chemistry. Sec **10** is broadly used as a standard sample to evaluate the ability of OSeCs to mimic the activity of Se-enzymes. In mechanistic studies, it is commonly used to monitor selenium metabolism. Its methylated form, MSC **7**, was found to act as an antioxidant agent and possess antiproliferative properties with respect to lung and thyroid cancer [12]. It was used as a wound healing stimulator [13], a building block, and a crosslinking initiator in synthesising peptides [14,15]. Selenodiglutathione **9** was also used to rebuild broken S-S bonds in peptides. Its physiological function allows it to be applied additionally as an efficient GPx mimetic and Trx substrate [16,17,18].

**Scheme 2:**
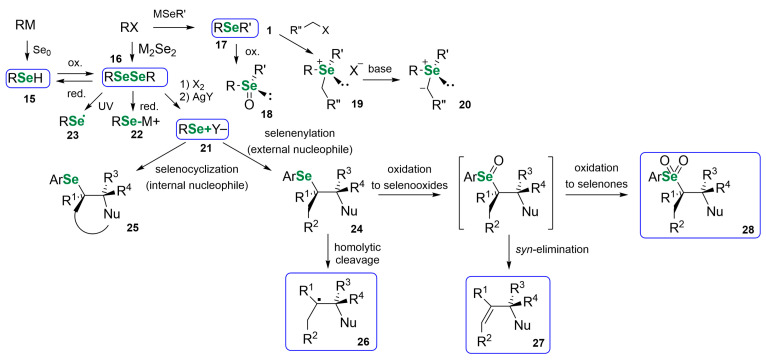
Synthesis and transformations of organoselenium reagents.

The reactivity of the selenium atom is broadly explored in chemical syntheses, enabling the design of efficient reagents, catalysts, and ligands for various reaction types. The advantages of Se-based transformations include the following: (1) mild reaction conditions (frequently fulfilling the rules of green chemistry); (2) diversified synthetic procedures to obtain OSeCs: selenol **15** (reaction of metal precursor and Se^0^), diselenide **16** (nucleophilic substitution of organic halide with M_2_Se_2_), and selenide **17** (reaction of RX with nucleophilic metal selenide MSeR); (3) transformation of selenide **17** to selenooxide **18**, selenonium salt **19,** and ylide **20** successfully used as chirality transfer agents; (4) simple cleavage of Se-Se bond and further transformation to electrophilic **21**, nuleophilic **22,** and radical **23** reagents; (5) facile introduction of the Se-moiety into the substrate compound (selenenylation **24** or selenocyclization product **25**); (6) efficient transformation of the intermediate Se-compound into a radical molecule **26** (homolytic cleavage of the Se-C bond), alkene **27** (selenooxide syn-elimination), and nucleophile precursor **28**; (7) broad possibility of application, e.g., drug design, materials science, and asymmetric synthesis as chiral catalysts and ligands (Scheme 2) [3,19,20,21,22,23,24,25].

In this context, constructing catalytically active molecules based on combining a chiral natural scaffold with a Se-moiety is an undeniably potent strategy for reagent design. In this review, we will present how incorporating a reactive selenium atom can enhance the reactivity of a natural compound or arm it with new, promising features. The collected material is divided to two sections: (2) naturally derived OSeCs as GPx-like catalysts, summarizing the most exploited field of application associated with the ability of OSeCs to act as oxygen transfer reagents mimicking the activity of GPx and (3) naturally derived OSeCs as reagents in organic synthesis, with subsections categorizing the gathered material according to the modified metabolites—amino acids and peptides (3.1), sugars (3.2), and terpenes (3.3).

## 2. Naturally Derived OSeCs as GPx-like Catalysts

Naturally derived organoselenium compounds (OSeCs) have emerged as promising oxygen transfer agents and glutathione peroxidase (GPx) mimetics due to their structural diversity, biocompatibility, and catalytic redox potential. Among the endogenous antioxidant defense systems, GPx enzymes play a pivotal role by catalyzing the reduction of hydrogen peroxide (H_2_O_2_) and organic hydroperoxides to water or corresponding alcohols using reduced glutathione (GSH) as the electron donor, which is concomitantly oxidized to glutathione disulfide (GSSG). As crucial components of the antioxidant defense system, they protect cells and tissues from oxidative damage that contributes to a multitude of diseases, e.g., cancer, diabetes, and neurodegenerative and cardiovascular disorders. [26,27,28,29,30,31]. The activity of GPx depends critically on the presence of the selenocysteine (Sec) residue at the active site, for which its selenol group (-SeH) possesses superior nucleophilicity and a lower pKa (~5.2) compared to cysteine’s thiol group, enhancing its reactivity under physiological conditions [32]. Substitution of this selenium center with sulfur (i.e., replacing Sec with Cys) drastically reduces enzymatic activity, confirming the essential catalytic function of selenium in redox biology [33,34]. Ebselen, a well-known synthetic organoselenium compound, is a benchmark GPx mimic. However, its poor aqueous solubility (logP ≈ 3.7) limits its bioavailability, necessitating specialized delivery strategies for biological evaluation [35].

In this context, various OSeCs derived from natural scaffolds such as amino acids, sugars, terpenes, steroids, or vitamins have been synthesized and studied for their GPx-like activity. Organoselenium compounds bearing amino acids, vitamins, and carbohydrate-based residues offer water solubility and potential for targeted delivery in biological systems. In contrast, terpene-derived organoselenium compounds benefit from conformational rigidity and stereocontrol, contributing to their catalytic performance [36]. These compounds effectively reduce peroxides and regenerate GSH, positioning them as valuable tools for modulating oxidative stress in biological systems. The exploration of naturally derived GPx mimetics thus holds great promise for developing antioxidant therapeutics with improved stability, selectivity, and pharmacological profiles.

Numerous review articles have summarized the state of the art in constructing OSeCs as efficient oxygen transfer reagents and GPx-like catalysts [25]. Herein, a summary of methods assessing the antioxidant potential of organoselenium compounds and the known naturally derived GPx mimetics will be presented.

### 2.1. Methods for Assessing GPx-like Antioxidant Activity

The table below (Table 1) summarizes the most commonly used methods for evaluating glutathione peroxidase (GPx) activity.

### 2.2. Examples of Organoselenium Compounds Mimicking GPx

Cholesterol, a well-known natural steroid, possesses unique structural features, such as a rigid framework with eight chiral centers and a high potential for structural derivatization [44,45,46]. In 2015, Braga et al. incorporated cholesterol into molecular systems by synthesizing selenides and diselenides. All synthesized diselenides exhibited strong GPx-like activity, with one compound, **29**, demonstrating activity 3.3 times higher than the reference compound, Ebselen [47].

The vitamin B6 family of pyridine derivatives, including pyridoxine, pyridoxal, and pyridoxamine, functions as cofactors in numerous enzymatic reactions involving amino acids, such as transamination, deamination, racemization, decarboxylation, and β-elimination [48]. In 2015, Singh and co-workers modified one of the vitamin B6 vitamers, pyridoxine, by incorporating a selenium atom in place of the methyl group at position 2 and a bromine atom at position 6. As a result of this substitution, derivative **30** exhibited enhanced antioxidant activity, showing a 2-fold increase compared to Ebselen [49].

Incorporating amino acids into chemical structures can significantly improve their biocompatibility and water solubility. Moreover, such modifications often enhance the biological activity and selectivity of the resulting compounds [50,51,52]. In 2018, Braga and Rocha synthesized a novel chiral diselenoamino acid derivative derived from phenylalanine and valine. Diselenide **31**, derived from *L*-valine, exhibited antioxidant activity comparable to (PhSe)_2_. The results indicated that the catalytic efficiency of the GPx mimetics described in this study is influenced by steric factors, which are affected by the length of the carbon chain separating the selenium atom from the amino acid residue and/or by the nature of the amino acid side chain [53]. In 2017, Ścianowski and co-workers synthesized a series of N-substituted benzisoselenazol-3(2H)-ones, functionalized at the nitrogen atom with amino acids, to improve Ebselen’s solubility. Among these derivatives, the compound **32** based on *L*-leucine was the most efficient peroxide scavenger, exhibiting 24-fold higher activity than ebselen [54].

Terpenes, widely occurring natural compounds in the plant kingdom, are characterized by a rich structural diversity and valuable properties. Traditionally employed as carriers of fragrance and flavor, they have also demonstrated significant pharmacological potential [55]. In recent years, in the research group led by Ścianowski, terpenes were effectively utilized as starting materials for the synthesis of a wide variety of chiral organoselenium compounds, including benzisoselenazol-3(2H)-ones [54,56], diselenides [55], phenylselenides [57], and selenides [58]. A notably high antioxidant potential was observed for benzisoselenazol-3(2H)-ones **33**,**34** and diselenides **35**,**36** containing the *N*-bornyl group. In the case of phenylselenides and selenides, high antioxidant potential was observed for *N*-pinanyl phenyl selenide **37** and *O*-menthyl selenide **38**, respectively.

Furthermore, within the same research group, a series of novel chiral benzisoselenazol-3(2H)-ones was synthesized, featuring nitrogen substitution with three different monoterpene moieties—*p*-menthane, pinane, and carane. The synthesized compounds were designed as pairs of enantiomers, epimers, and regioisomers to further evaluate how the specific three-dimensional arrangement of substituents influences biological activity. The best antioxidant activity was observed for *N*-substituted benzisoselenazolones **39**,**40** with α-pinane skeletons. Interestingly, as anticipated, the compounds exhibited different antiproliferative capacity on human promyelocytic leukemia HL-60 cell lines. A recent publication by João P. Telo et al. reported a one-pot reaction of the natural monoterpenoid (−)-carvone with selenium bromide, yielding menthoselenophenone as the main product, along with minor amounts of phenolic by-products. A series of derivatives was also synthesized: an α,α-dimer, an oxime, and its Beckmann rearrangement product, lactam. Among the compounds obtained, dimer **41** exhibited the highest antioxidant activity [59].

Selenoneine, a selenium analogue of ergothioneine, is naturally found as the predominant organic selenium species in marine fish’s blood and muscle tissue, such as bluefin tuna and mackerel. This compound originates from marine food chains, being biosynthesized by microorganisms and bioaccumulated in predatory fish. It is then transferred to humans through dietary intake, which functions as a potent antioxidant [60]. For this reason, in 2023, Gaucher, da Silva Junior et al. focused on synthesising a series of molecules based on the selenoneine structure. Notably, compound **42** bearing a trifluoromethyl group exhibited GPx-mimetic activity similar to that of Ebselen [61]. The table below summarizes examples of all the naturally derived organoselenium compounds described above that exhibit the highest glutathione peroxidase (GPx)-mimicking activity and the methods used to evaluate this antioxidant activity (Table 2).

## 3. Naturally Derived OSeCs as Reagents in Organic Synthesis

The development of chiral reagents, catalysts, or ligands is highly valuable in modern organic synthesis, the total synthesis of natural products, and medicinal chemistry, where the enantioselectivity of the process is a major issue in many cases. The fixed stereochemistry of natural compounds like amino acids, sugars, and terpenes induced their broad applicability as synthetic tools in stereoselective organic synthesis. The additional incorporation of a Se-moiety has gained much attention, supported by the unique reactivity of the selenium atom. Until now, several organoselenium reagents (OSeRs) based on natural products like amino acids, sugars, and terpenes have been obtained and efficiently applied in various organic transformations.

### 3.1. Se Amino Acids and Peptides

**Scheme 3:**
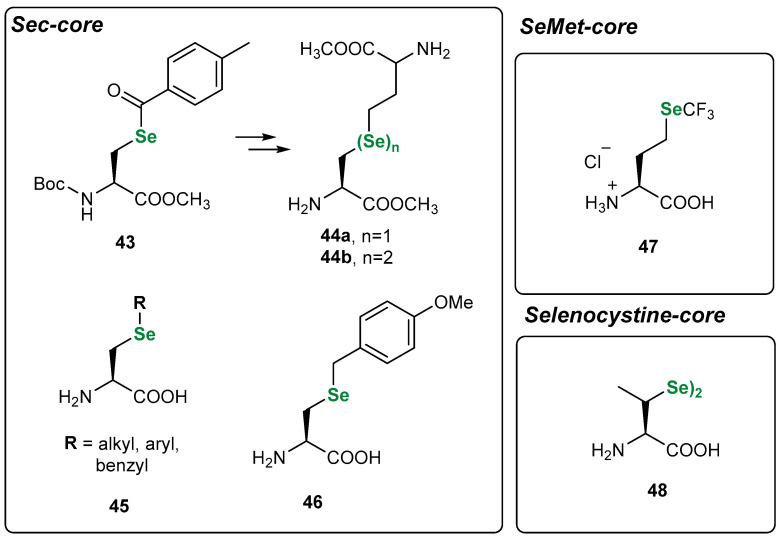
Modification of Se amino acids **43**–**48**.

Only 22 amino acids are what nature needed to form peptides fundamental to life. This group of compounds is considered essential primary metabolites and potential drug candidates and chiral reagents/catalysts in modern organic synthesis [62]. Some natural amino acids, as presented in Scheme 1, also possess an incorporated selenium atom, which makes them essential for several biochemical processes. Artificial Se amino acids can be synthesized by modifying the natural Se-metabolites or attaching a selected Se-moiety to the amino acid backbone. The catalytically potent Sec **1** was most thoroughly investigated—synthetic procedures and modification protocols were summarized in the review presented by Wessjohan [63]. More recent transformations included the conjugation of different aliphatic and aromatic substituents with the reactive selenol group. *N*-Boc-protected Sec **43**, obtained from the *L*-serine methyl ester, was further modified into selenolanthionine **44a** and selenocystine **44b**, derivatives efficiently applied to synthesize Se-peptides [64]. Other SeH-conjugates **45** and **46** were proven to inhibit human cytochrome P450 [65,66] and possess anticancer properties, respectively [67]. Trifluoromethyl derivative of selenomethionine **47**, synthesized from *N*-(tert-butoxycarbonyl)-*L*-aspartic acid *tert*-butyl ester by a seven-step procedure, was also evaluated as a cytotoxic agent towards colon cancer [68]. Lastly, a methylated form of selenocystine **48** was obtained to evaluate the release and bioavailability of the selenium atom from unnatural amino acids. As a result, Se accumulation in rats’ blood and liver was observed [69] (Scheme 3).

**Scheme 4:**
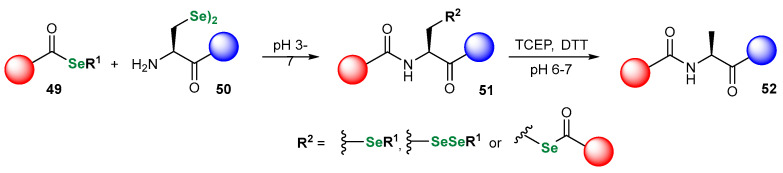
Utility of Se amino acids in NCL.

The possibility of Sec-modification enables the functionalization of proteins at these amino acid residues [70]. Modified proteins are essential for discovering biologics and investigating molecular mechanisms in biological processes. Thus, methods for precise peptide synthesis and functionalization are highly needed. The native chemical ligation (NCL), a tool for protein preparation, can occur 1000-fold faster for Sec than for the corresponding Cys residues. As the occurrence of Sec is also very low (>0.001% of protein residues) in comparison to Cys (about 0.3%), the reactions can be performed more selectively, leading to the total synthesis of proteins with particular modifications. An example of NCL utilizing Se-reagents is presented in Scheme 4. The Se-functionality of selenoester **49**, an acyl group transfer agent, undergoes a nucleophilic substitution performed with Sec-peptide **50**. Later, the undesired Se-moiety of **51** can be eliminated and transformed to alanine through anaerobic deselenization by tris (2-carboxyethyl) phosphine and DTT [71,72].

**Scheme 5:**
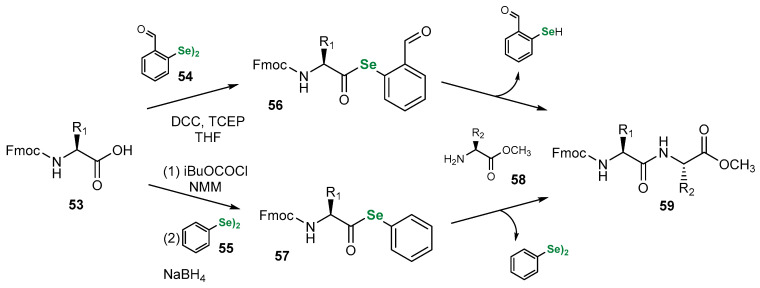
Se-esters as acyl group transfer agents.

Other examples of using selenoesters as acyl group transfer agents in synthesizing peptides are presented in Scheme 5. Reagents **56** and **57** were generated from Fmoc-protected amino acids **53** and diphenyl diselenides **54** and **55**. After forming the final amino acid **59**, the eliminated organoselenium fragments can be efficiently regenerated [73,74].

The applicability of selenium in peptide chemistry was recently summarized in a review published by Stefanowicz et al. [75]. The presence of the Se-moiety enables the synthesis and post-synthetic modification of peptides based on the following: (a) formation of diselenide bridges (protein folding); (b) construction of selenolanthionie linkages (cyclisation and stapling); (c) and susceptibility of the diselenide bond to UV-irradiation, facilitating photochemical transformations [76,77,78,79].

### 3.2. Se-Sugars

**Scheme 6:**
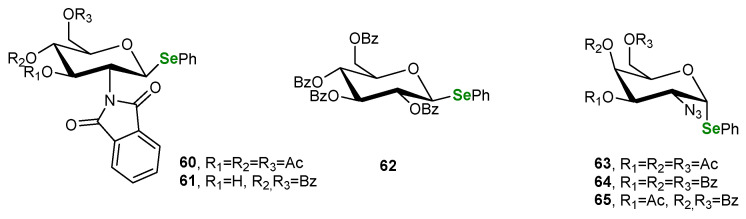
Phenylselenoglycosides as glycosyl group transfer agents.

The chemistry of carbohydrates is one of the most relevant research fields in organic synthesis. These polyfunctional compounds possess several advantages: They can be diversely manipulated to form new reactive centers or coordination sites; their attachment to other derivatives can enhance water solubility; as molecules vital for a multitude of biochemical processes, they can be considered potential pharmacophores for bioactive compounds [80,81]. A recent review by Santi et al. summarizes the synthesis of bioactive Se-containing sugars [82]. When considering the incorporation of selenium for OSeRs design, the phenylselanyl group dominates as the Se-sugar reactive center. Since the work of Pinto et al. [83] and the utility of phenylselenoglycosides as glycosyl group donors, this application route is continuously being fed with new selenosugar-based protocols. The RSePh derivatives are mainly used as glycosyl group transfer agents. Examples of phenylselenoglycosides **60**–**65**, efficiently applied in the *O*-glycosylation reaction, are presented in Scheme 6 [84,85,86].

**Scheme 7:**
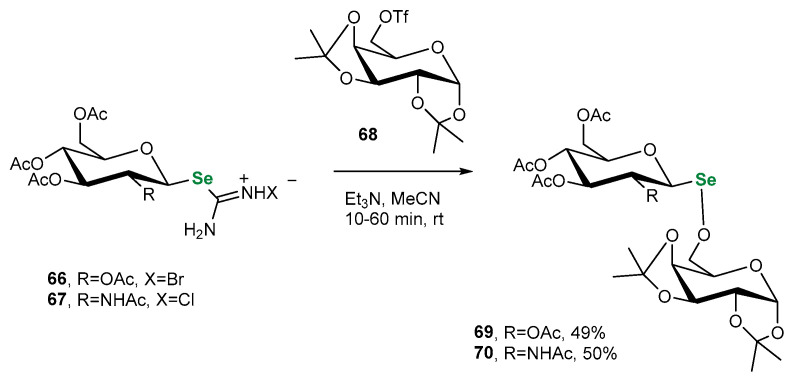
Isoselenuronium reagents in the synthesis of Se-disaccharides.

The construction of naturally derived compounds using Se-sugar reagents was also performed utilizing different types of Se-moieties. Szilayi et al. obtained a series of isoselenuronium bromides **66** or chlorides **67** that were further transformed to variously substituted Se-glycosides [87]. Under mild reaction conditions, the nucleophilic substitution with monosaccharide triflate **68** enabled the formation of Se-disaccharides **69** and **70** in good yields (Scheme 7).

**Scheme 8:**
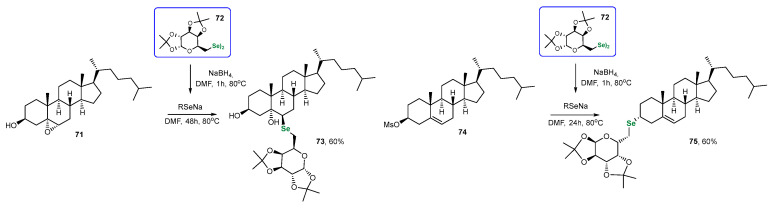
Synthesis of steroidal selenoglycoconjugates.

Next, Ludtke and Wessjohann applied sugar diselenide **72** to synthesize steroidal selenoglycoconjugates **73** and **75** [88]. The reaction was performed with nucleophilic Se-reagent generated by the treatment of compound **72** with sodium borohydride. The further ring opening of steroidal epoxide **71** or the nucleophilic substitution of steroidal mesylate **74** furnished products **73** and **75**, respectively (Scheme 8). The carbohydrate scaffold was further deprotected by the treatment with trifluoroacetic acid/methanol (9:1).

**Scheme 9:**
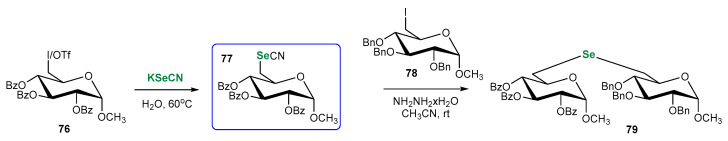
Synthesis of glycosyl selenocyanates in on-water conditions.

Lately, Misra et al. designed a reaction of appropriate glycosyl iodides or triflate **76** with potassium selenocyanate in water, which yielded a series of RSeCN-type reagents **77**. These building blocks were further applied in synthesizing Se-pseudodisaccharide **79** (Scheme 9) [89].

### 3.3. Organoselenium Reagents Derived from Monoterpenes

Considering all enantiopure compounds creating the natural chiral pool, terpenes, with about 55,000 different structures, are the dominating contributors [90]. Depending on the number of isoprene units (C_5_)n that build up the isoprenoid skeleton, these derivatives are divided into mono- (C_10_), sesqui (C_15_), di- (C_20_), and polyterpenes (C_n≥40_) [91]. The physicochemical properties of monoterpenes enable their feasible isolation from natural feedstocks by steam distillation. Bio-wastes from the orange juice and paper industry are the main sources for isoprenoid acquisition. Broad natural abundance, exceptional diversity of carbon skeletons, various functional groups, and stereogenic centers grounded their position as chirality transfer agents and building blocks in various organic transformations, stereoselective synthesis, and total synthesis of natural products [92].

Until now, the combination of a chiral monoterpene scaffold with a reactive selenium moiety has been well explored in the design of reagents for asymmetric transformations. Since the pioneering work of Sharpless—the synthesis of allylic alcohol linalool through the [2,3]-sigmatropic rearrangement of an allylic Se(IV) derivative—a multitude of chiral reactions have been designed utilizing Se-reagents constructed on the monoterpene skeleton. This subsection will be divided according to the appropriate transformation: monoterpene-based OSeRs in [2,3]-sigmatropic rearrangements (3.3.1); monoterpene-based OSeCs as electrophilic reagents (3.3.2); monoterpene-based OSeRs in asymmetric epoxidation and cyclopropanation (3.3.3); and other asymmetric transformations utilizing chiral monoterpene-based OSeRs (3.3.4).

#### 3.3.1. Monoterpene-Based OSeRs in [2,3]-Sigmatropic Rearrangements

**Scheme 10:**
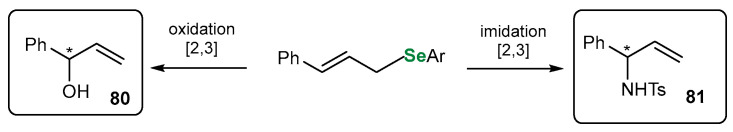
The [2,3]-sigmatropic rearrangement of Se(IV) species to afford chiral alcohols and amines.

The discovery of [2,3]-sigmatropic rearrangement through allylic selenooxides, with subsequent selenooxide elimination, has popularised the utility of OSeCs in general organic synthesis. The reaction can be performed under mild conditions, much faster than with corresponding sulfoxides. When considering stereoselective versions, it can lead to high enantiomeric excess of the final products. The Se-reagent can generally undergo intermolecular rearrangement after oxidation or imidation via corresponding selenimides. These highly valuable reactions efficiently route chiral alcohol **80** and amine **81** (Scheme 10).

**Scheme 11:**
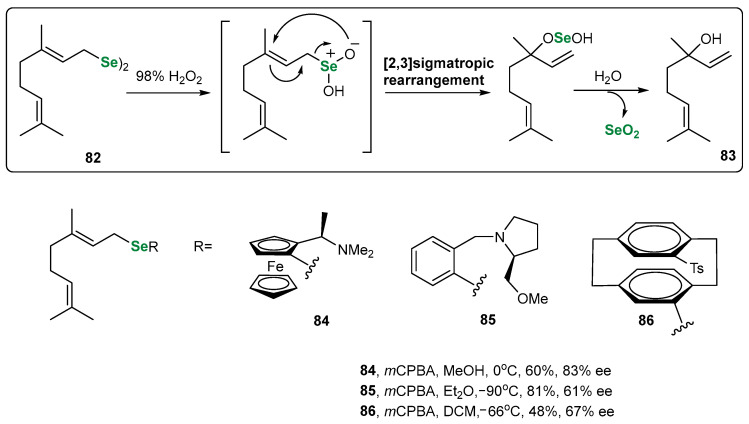
The [2,3]-sigmatropic rearrangement of geranyl diselenide **82**.

The first transformation of a monoterpene diselenide **82** to the corresponding allylic alcohol **83** was presented by Sharpless in 1972 [93,94,95]. Later, different types of chiral geranyl selenides, e.g., **84**–**86**, were also successfully evaluated as precursors for the [2,3]-shift to afford compound **83** [96,97,98] (Scheme 11).

**Scheme 12:**
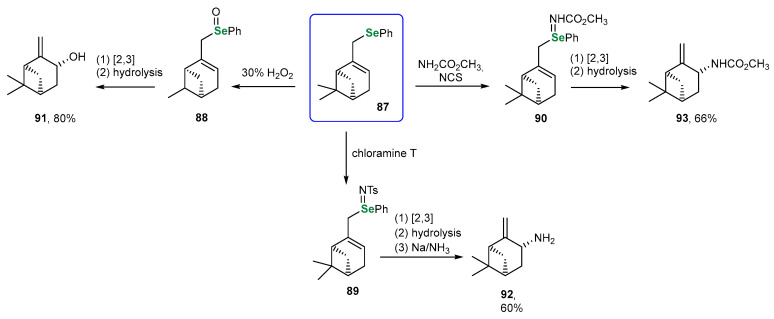
The [2,3]-sigmatropic rearrangement of pinanyl selenooxide **88** and selenoimides **89** and **90**.

Several application examples of monoterpene OSeCs—monocyclic ((+)-carvotanacetyl, (−)-carvyl, perillyl), bicyclic (carane, pinane), and acyclic (geranyl, neryl) phenylselenides—in the synthesis of chiral amines and alcohols through [2,3]-shift were presented in the early 2000s by Ścianowski and co-workers [99,100,101,102,103]. The synthetic procedure is presented using (+)-10-phenylseleno-2-pinene **87** as an example. Treatment of precursor **87** with hydrogen peroxide, NCS, and chloramine-T led to selenooxide **88** and selenoimides **89** and **90**, respectively. Subsequent rearrangement and hydrolysis furnished the final chiral products **91**–**93** (Scheme 12).

**Scheme 13:**
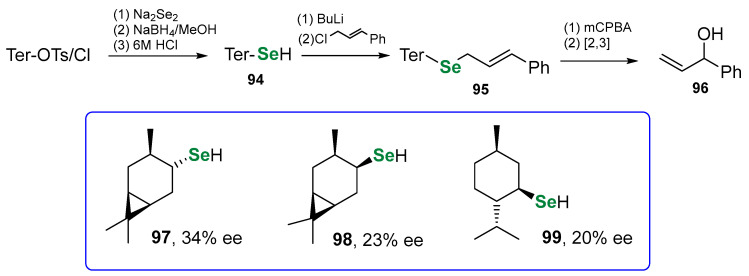
Terpene selenols as [2,3]-sigmatropic rearrangement precursors.

Similarly, terpenyl selenols were tested as [2,3]-shift precursors of chiral allylic alcohols. The obtained RSeH **94** was transformed to the corresponding allylic selenide **95** by treatment with butyl lithium and appropriate allylic chloride [104]. The oxidation with *m*CPBA following [2,3]-SR furnished the final chiral product **96** with moderate enantioselectivities (examples **97**–**99**, Scheme 13).

**Scheme 14:**
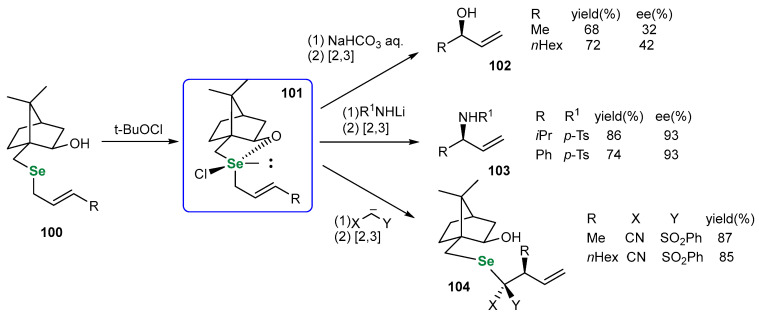
Synthesis of chiral alcohols, amines, and selenides from chloroselenourane **101**.

Koizumi and co-workers also efficiently used terpene selenonium ylides as [2,3]-sigmatropic rearrangement precursors [105,106,107]. Allylic selenide **100** was transformed into allylic chloroselenurane **101**, which, via the formation of corresponding selenooxides, selenimines, and aforementioned selenonium ylides, underwent rapid [2,3]-shifts to form chiral alcohol **102**, amine **103**, and selenide **104**, respectively (Scheme 14).

#### 3.3.2. Monoterpene-Based OSeCs as Electrophilic Reagents

The 1,2-addition to alkenes performed with electrophilic organoselenium reagents is a very well-examined field of research. The mechanistic features of this process include the reaction of RSe^+^X^−^ (e.g., X = Cl, OTf, OSO_3_H) with alkene and the subsequent formation of a seleniranium ion. Further inter- or intramolecular ring opening with selected nucleophiles leads to acyclic or cyclic products [108]. Attachment of chiral moieties like terpenes to the reactive selenium center facilitates an asymmetric process. Back and co-workers performed one of the first enantioselective additions using a C_3_-camphor-based diselenide [109,110]. Methoxyselenenylation of *trans*-dec-5-ene with triflates **105** and **106** gave excellent *d.r.* values. The presence of an oxime enhances diastereoselectivity due to the coordination of the hydroxyl group with the selenium atom. Modified C_3_-camphor reagents were also applied in the cyclisation of unsaturated alcohols and carboxylic acids. Chloride **107** yielded the cyclic ether with *d.r.* 84:16 [111,112,113,114,115]. Later, Ścianowski et al. synthesized and transformed other terpene diselenides derived from carane, *p*-menthane, and pinane into selenenyl triflate analogs. The 1,2-addition to styrene with the highest diastereoselectivity was observed for hydroxycarane derivative **108**. The selenocyclization of *o*-allylphenol bicyclic myrtenyl triflate **109** furnished the final benzofuran with *d.r.* 86:14 [116,117] (Table 3).

**Scheme 15:**
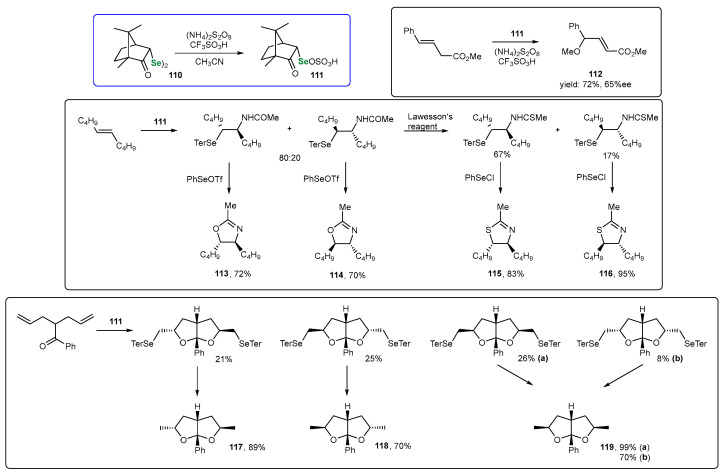
The utility of camphor selenenyl sulfate **111** in asymmetric selenenylation–elimination transformations.

The group of Tiecco also performed several elegant asymmetric selenenylation–elimination transformations. The camphor diselenide **110** was converted to the corresponding sulfate **111** by the treatment with ammonium persulfate and triflic acid. The further utility of reagent **111** included the synthesis of (a) allylic ether **112** [118,119]; (b) oxazolines **113** and **114** and thiazolines **115** and **116** [120,121]; and (c) perhydrofuro [2,3-b]furans **117**–**119** [122] (Scheme 15).

The same group had also further exploited the utility of camphor diselenide **110** through its transformation to camphor selenenyl triflate in the synthesis of 1,6-dioxaspiro[4,4]nones [123] and (*R*)-camphorselenoacetone or methyl (*R*)-camphorselenoacetate as nucleophilic reagents in TiCl_4_-mediated aldol condensation [124,125].

#### 3.3.3. Monoterpene-Based OSeCs in Asymmetric Epoxidation and Cyclopropanation

**Scheme 16:**
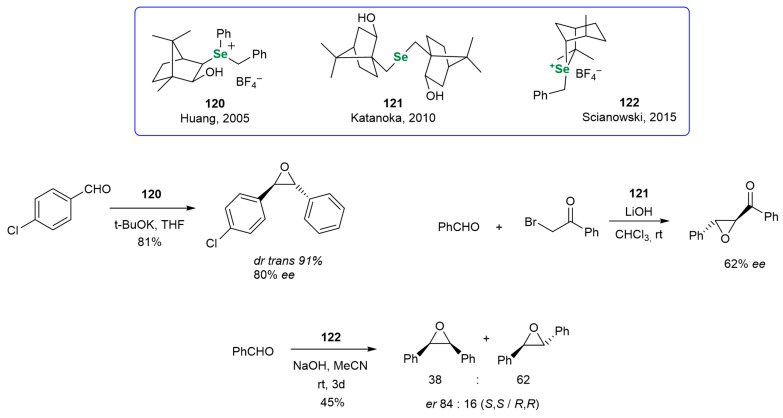
Synthesis of chiral epoxides using terpenyl OSeRs [126,127,128].

The design of efficient protocols to construct small rings like epoxides and cyclopropanes is crucial because of their exceptional reactivity and applicability as important building blocks. To date, several research groups have taken up the task of using terpenyl OSeRs in asymmetric epoxidation and cyclopropanation reactions. For the enantioselective synthesis of oxiranes, the camphor scaffold was modified by Huang [126] and Katanoka [127]. C_3_-camphor selenonium salt **120** and C_2_-camphor selenide **121** were obtained and applied to synthesize diphenyl oxiranes (up to 80% *ee*) and the Darzens reaction (up to 62% *ee*), respectively. Later, Midura and Ścianowski designed a series of bicyclic and acyclic terpenyl selenonium salts for the asymmetric epoxidation of benzaldehyde. The best result, acquired for (+)-limonene derivative **122**, gave an enantiomeric ratio of 84:16 [128] (Scheme 16).

**Scheme 17:**
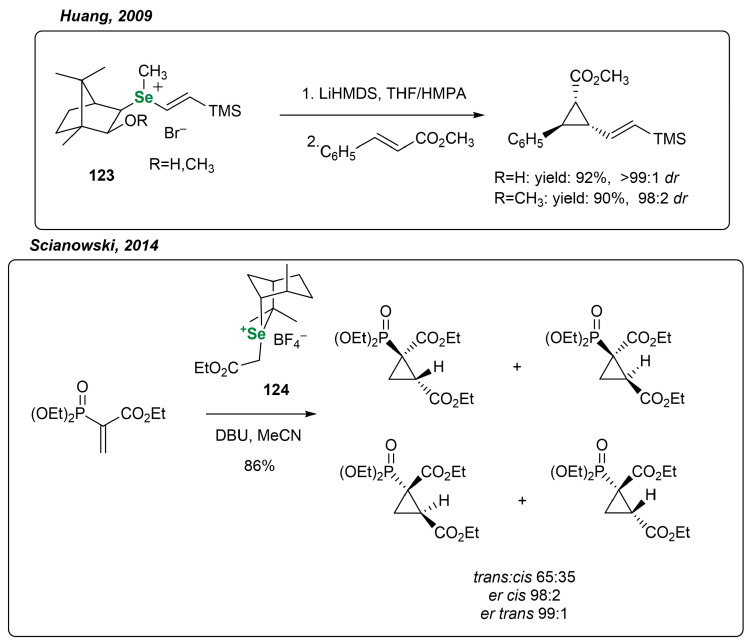
Synthesis of cyclopropanes using terpenyl OSeRs [129,130].

Application of terpenyl OSeRs in the synthesis of cyclopropanes covers two protocols presented by Huang in 2009 [129] and Ścianowski in 2014 [130] (Scheme 17). The first asymmetric synthesis of trimethylene derivatives performed with C_3_-camphor selenonium ylides **123** gave promising results with diastereomeric ratios up to 99:1. Later, excellent enantioselectivity (*er cis* 98:2, *er trans* 99:1) for the cyclopropanation of vinylphosphonate with (+)-limonene-derived selenonium ylides **124** confirmed the validity of using these types of reagents in the synthesis of small rings.

#### 3.3.4. Other Transformations Utilizing Chiral Monoterpene-Based OSeCs

**Scheme 18:**
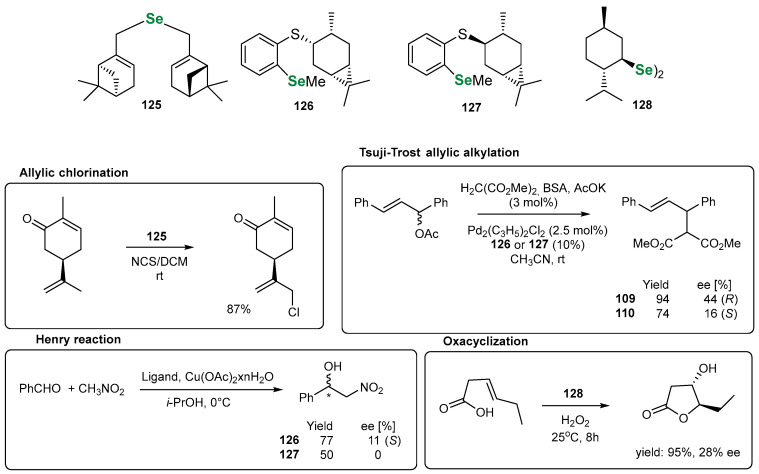
Miscellaneous transformations utilizing chiral monoterpene-based OSeCs.

In addition to the well-established ways of utilizing naturally derived organoselenium reagents, new research protocols are emerging, expanding the scope of known reactions susceptible to Se-based transformations and catalysis. Examples of terpenyl OSeCs applied in different types of reactions are presented in Scheme 18. Allylic chlorination performed in the presence of pinene selenide **125** furnished the final chlorides in excellent yields [131]. Methylselenides with *o*-(*S*-caranyl)- and *o*-(*S*-isocaranyl) moieties **126** and **127** were evaluated as catalysts in the Tsuji–Trost allylic alkylation and Henry reaction [132]. Both reactions led to good yields but low enantioselectivities. A similar result was observed for the oxacyclization of alkenoic acids with the utility of dimenthyl diselenide **128** (yield: 95%, 28% ee) [133].

## 4. Conclusions

Natural metabolites serve as valuable starting materials in the design of organoselenium reagents and catalysts due to their inherent structural diversity and chirality. Numerous selenides, diselenides, selenols, selenonium salts, and ylides have been successfully synthesized using frameworks derived from amino acids, sugars, terpenes, and steroids. These compounds not only demonstrate considerable synthetic potential but also possess significant antioxidant properties and promising biological activity, aligning them closely with the goals of medicinal chemistry. Despite these advantages, the application of such selenium-containing reagents in modern organic synthesis remains underexploited. Their broader application could offer distinct benefits, including improved selectivity, biocompatibility, and access to enantiomerically pure products. This review highlights both the current achievements and the untapped potential of these compounds in synthesis, catalysis, and biomedical applications. Further exploration in this field may lead to the development of novel, efficient, and sustainable methodologies with broad implications for drug discovery and therapeutic innovation.

## Figures and Tables

**Table 1:** Methods for evaluating glutathione peroxidase (GPx) activity.

Methods for Assessing GPx-like Antioxidant Activity		Methods for Assessing Radical-Trapping Antioxidant Activity
**GSH/GR Coupled Assay (Enzymatic Method)** [37]		**ABTS Assay** [38]
The glutathione reductase (GR)-coupled assay was the pioneering indirect method for assessing GPx-mimic activity, created by Wilson et al. In this process, the GR enzyme utilizes the cofactor NADPH (β-nicotinamide adenine dinucleotide 2′-phosphate) to catalyse the conversion of oxidised glutathione (GSSG), produced during the catalytic reaction, back to its reduced form (GSH). The initial rates of NADPH reduction (νo) are measured using UV spectroscopy at a wavelength of 340 nm. The assay solution is prepared by mixing a potassium phosphate buffer, EDTA, sodium azide, GR, and a suitable amount of the test compound. The subsequent addition of H_2_O_2_ starts the reaction. The half and overall equations for the occurring reactions are as follows (Equations (1)–(3)):		In the method introduced by Shaaban et al., the antioxidant activity of organoselenium compounds is evaluated based on their capacity to decolourise ABTS (2,2′-azino-bis(3-ethylbenzothiazoline-6-sulfonic acid) radicals. The corresponding radical-scavenging activity is determined by measuring the reduction in absorbance at 734 nm.
$2\mathrm{GSH}+H_{2}O_{2}\;\overset{\begin{array}{c} GSH\\ peroxidase \end{array}}{\to }\mathrm{GSSG}+2H_{2}O$	(1)	
$GSSG+\mathrm{NADPH}+H^{+}\;\;\overset{\begin{array}{c} GSH\\ reductase \end{array}}{\to }2\mathrm{GSH}+{\mathrm{NADP}}^{+}$	(2)	
$H_{2}O_{2}+\mathrm{NADPH}+H^{+}\;\to {\mathrm{NADP}}^{+}+2H_{2}O$	(3)	
**PhSH assay** [39]		**DPPH assay** [40]
In this method, proposed by Iwaoka and Tomoda, benzenethiol (PhSH) is utilized as a GSH substitute. The reduction of hydrogen peroxide in the presence of PhSH, which leads to the concurrent production of diphenyl disulfide (PhSSPh), is evaluated using various methods: a. spectrophotometric analysis measures the increase in UV absorption at 305 nm resulting from the formation of PhSSPh; b. HPLC analysis determines the quantity of PhSSPh produced by measuring the time needed for 50% of PhSH to convert to PhSSPh (t_1/2_ values), calculated from the peak areas at different time intervals. The equation for the described reaction is as follows (Equation (4)):		Shaaban et al. presented a straightforward method for evaluating the radical scavenging activities of organoselenium compounds and nutritional products. The antioxidant capacity of a compound is determined by its ability to convert the stable DPPH·radical (which appears purple in methanol) to DPPH (colourless), as indicated by a reduction in absorbance at 517 nm.
$2PhSH+H_{2}O_{2}\overset{Se\text{-}catalyst}{\to }PhSSPh+2H_{2}O$	(4)	
**DTTred/DTTox NMR assay** [41]		**HPLC Lipid Peroxidation assay** [42,43]
The GPx-like antioxidant activity of compounds can be assessed using the protocol developed by Iwaoka et al. In this approach, the organoselenium catalyst reduces H_2_O_2_ and is subsequently regenerated in the presence of dithiothreitol (DTT^red^). The kinetics of this reaction are analysed using ^1^H NMR spectroscopy in either CD_3_OD or D_2_O. Signals corresponding to the disulfide (DTT^ox^) produced at specific intervals are recorded. The chemical equation that describes the reaction is as follows (Equation (5)):		To evaluate the antioxidant properties of organochalcogen compounds, Engman et al. routinely employed azo-initiated peroxidation of linoleic acid and its derivatives. A recent study described a refined experimental model for determining inhibition times (T_inh_) and rates of peroxidation inhibition (R_inh_) within a biphasic system. In this modified approach, the lipid phase contained linoleic acid and the test antioxidant, while the aqueous phase included a water-soluble co-antioxidant, such as N-acetylcysteine (NAC), capable of regenerating the active antioxidant species. The reaction mixture, chlorobenzene containing linoleic acid and the antioxidant, was vigorously stirred at 42 °C with the NAC solution. Peroxidation was initiated by 2,2′-Azobis(2,4-dimethylvaleronitrile) (AMVN), and the formation of conjugated dienes was monitored by HPLC with UV detection at 234 nm. The inhibition rate (R_inh_) was subsequently calculated using least-squares analysis of absorbance versus time data.
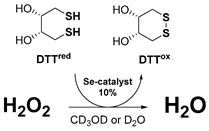	(5)	

**Table 2:** Examples of naturally derived organoselenium compounds with the highest glutathione peroxidase (GPx)-mimicking activity.

The Structure of the Compound	Origin	Method for Assessing GPx-like Antioxidant Activity	Author
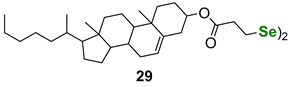	Cholesterol-derived diselenide	PhSH assay	Braga et al.
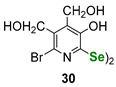	Pyridoxine (vitamin B6)-derived diselenide	GSH/GR coupled assay	Singh et al.
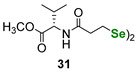	Amino acid (L-valine)-derived diselenide	PhSH assay	Braga, Rocha et al.
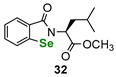	Amino acid (L-leucine)-derived benzisoselenazolone	DTT^red^/DTT^ox^ NMR assay	Ścianowski et al.
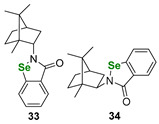 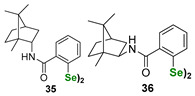	Terpene (camphane)-derived benzisoselenazolones and diselenides	DTT^red^/DTT^ox^ NMR assay	Ścianowski et al.
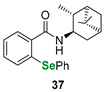	Terpene (pinene)-derived phenylselenide	DTT^red^/DTT^ox^ NMR assay	Ścianowski et al.
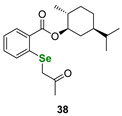	Terpene (O-menthyl)-derived selenide	DPPH assay	Ścianowski et al.
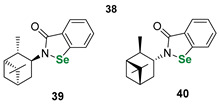	Terpene (pinene)-derived benzisoselenazolones	DTT^red^/DTT^ox^ NMR assay	Ścianowski et al.
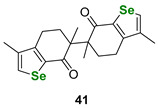	Terpene carvone-derived selenophenes	DTT^red^/DTT^ox^ NMR assay	João P. Telo et al.
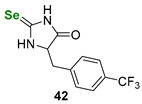	Selenoneine-derived selenohydantoin	GSH/GR coupled assay	Gaucher, da Silva Júnior et al.

**Table 3:** Electrophilic terpenyl OSeRs applied in the asymmetric 1,2-addition to alkenes.

Reagent	Substrate	Product	Yield [%], *d.r.*	Author
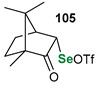	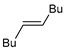	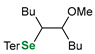	88, 94:6	Back et al.
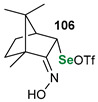	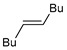	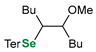	88, 98:2	Back et al.
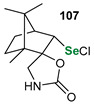	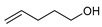	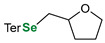	87, 84:16	Back et al.
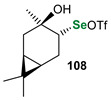	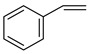	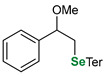	54, 90:10	Ścianowski et al.
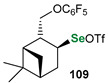	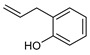	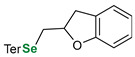	49, 86:14	Ścianowski et al.

## Data Availability

Not applicable.
